# Characterization of the interaction between the HIV-1 Gag structural polyprotein and the cellular ribosomal protein L7 and its implication in viral nucleic acid remodeling

**DOI:** 10.1186/s12977-016-0287-4

**Published:** 2016-08-11

**Authors:** Hala El Mekdad, Emmanuel Boutant, Hassan Karnib, Marina E. Biedma, Kamal Kant Sharma, Iuliia Malytska, Géraldine Laumond, Marion Roy, Eléonore Réal, Jean-Christophe Paillart, Christiane Moog, Jean Luc Darlix, Yves Mély, Hugues de Rocquigny

**Affiliations:** 1 Laboratoire de Biophotonique et Pharmacologie, UMR 7213 CNRS, Faculté de Pharmacie, Université de Strasbourg, 74, Route du Rhin, 67401 Illkirch Cedex, France; 2 Fédération de Médecine Translationnelle de Strasbourg (FMTS), INSERM U1109, Université de Strasbourg, 3 rue Koeberlé, 67000 Strasbourg Cedex, France; 3 Architecture et Réactivité de l’ARN, CNRS, Institut de Biologie Moléculaire et Cellulaire, Université de Strasbourg, 15 rue René Descartes, 67084 Strasbourg Cedex, France

**Keywords:** HIV, Gag, RPL7, Interaction, Chaperone activity, Nucleocapsid

## Abstract

**Background:**

In HIV-1 infected cells, the integrated viral DNA is transcribed by the host cell machinery to generate the full length HIV-1 RNA (FL RNA) that serves as mRNA encoding for the Gag and GagPol precursors. Virion formation is orchestrated by Gag, and the current view is that a specific interaction between newly made Gag molecules and FL RNA initiates the process. This in turn would cause FL RNA dimerization by the NC domain of Gag (GagNC). However the RNA chaperoning activity of unprocessed Gag is low as compared to the mature NC protein. This prompted us to search for GagNC co-factors.

**Results:**

Here we report that RPL7, a major ribosomal protein involved in translation regulation, is a partner of Gag via its interaction with the NC domain. This interaction is mediated by the NC zinc fingers and the N- and C-termini of RPL7, respectively, but seems independent of RNA binding, Gag oligomerization and its interaction with the plasma membrane. Interestingly, RPL7 is shown for the first time to exhibit a potent DNA/RNA chaperone activity higher than that of Gag. In addition, Gag and RPL7 can function in concert to drive rapid nucleic acid hybridization.

**Conclusions:**

Our results show that GagNC interacts with the ribosomal protein RPL7 endowed with nucleic acid chaperone activity, favoring the notion that RPL7 could be a Gag helper chaperoning factor possibly contributing to the start of Gag assembly.

**Electronic supplementary material:**

The online version of this article (doi:10.1186/s12977-016-0287-4) contains supplementary material, which is available to authorized users.

## Background

In HIV-1 infected cells, the integrated viral DNA is transcribed by the host cell machinery generating the full-length viral RNA (also referred to FL RNA), a large fraction of which undergoes splicing to give rise to single and multi-spliced viral mRNAs [1]. Once exported from the nucleus to the cytoplasm, the FL RNA can be recruited by active ribosomes to direct synthesis of the Gag and GagPol precursors [2]. In infected cells, Gag orchestrates virion formation in a process that necessitates two platforms. The first one is thought to be the FL RNA acting as a scaffold for Gag oligomerization upon binding. The second platform corresponds to the phospholipid bilayer of the plasma membrane in which Gag-FL RNA complexes are progressively anchored by the N-terminus of the Gag matrix (MA) domain [3, 4].

The Gag polyprotein precursor is formed of several domains that are the matrix (MA), capsid (CA), nucleocapsid (NC) and p6 as well as the spacer peptides p2 and p1 flanking NC [5, 6]. The FL RNA has a long 5′ untranslated region (UTR) containing a specific packaging signal composed of four stem-loops that mediate the binding of the NC domain of Gag (GagNC) and subsequently the formation of a dimeric FL RNA genome present in the viral particle [7–15]. This FL RNA dimerization is driven by the nucleic acid chaperone activity of GagNC [16–19] that directs structural rearrangements of nucleic acid molecules which rapidly reach their most stable structure [20, 21]. However, it was found that GagNC chaperone activity was low as compared to the mature NCp7 protein present in infectious virions [22–24]. Along this line, GagNC was also shown to hardly anneal primer tRNA^Lys,3^ to the Primer Binding Site (PBS), thus causing a profound defect in viral DNA synthesis [25–27].

This prompted us to look for host chaperoning factors cooperating with GagNC. Interestingly, several host proteins potentially interacting with Gag have been identified [28–30]. One of these proteins is RPL7 which is located at the surface of the large (60S) ribosomal subunit [31, 32] (referred in the recent nomenclature to as RPL30 [33]). RPL7 interacts with RNA [34, 35] and is involved in ribosome biogenesis [36] and the regulation of mRNA translation [37]. Since several *E. coli* ribosomal proteins behave as RNA chaperones [38, 39], we hypothesized that RPL7 may be endowed with chaperone activity and may thus cooperate with Gag to direct nucleic acid rearrangements.

By means of yeast two-hybrid and co-immunoprecipitation experiments, we confirmed the interaction between Gag and RPL7 and showed that both the N- and C-termini of RPL7 as well as the NC domain of Gag are the main determinants for this interaction. Also, the Gag–RPL7 interaction seems to be independent of cellular RNA and on Gag assembly suggesting that Gag monomers or small Gag oligomers could recruit RPL7 during the translation process. Using an in vitro model assay [19], RPL7 was found to exhibit a higher nucleic acid annealing activity than Gag, and that both proteins can act in concert to direct the rapid annealing of complementary RNAs and DNAs. Taken together, our data suggest that RPL7 may assist GagNC at the very beginning of assembly by augmenting its chaperone activity.

## Results

### HIV-1 Gag interacts with the cellular ribosomal protein RPL7

**Fig. 1:**
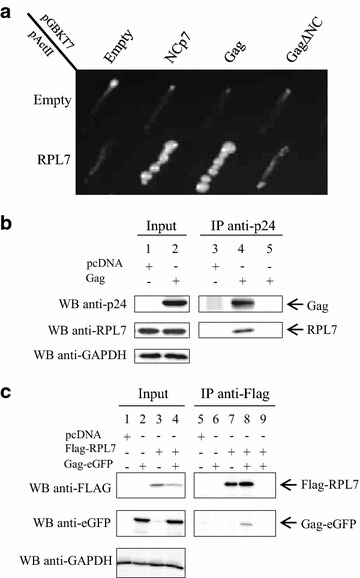
HIV-1 Gag interacts with human ribosomal protein RPL7. **a** Photograph of a plate with diploid yeast cells expressing Gal4BD alone or fused to either NCp7, Gag or GagΔNC (pGBKT7 construct) and Gal4AD alone or in fusion to RPL7 (pActII construct). Yeasts were plated on minimal media lacking histidine, leucine and tryptophan and incubated during 6 days at 30 °C. Growth in the absence of histidine reveals the interaction between the hybrid proteins. Note that NCp7 and Gag interact with RPL7 while marginal interaction was observed between Gag∆NC and RPL7. **b** Gag co-precipitates with endogenous RPL7. Lysates from HeLa cells transfected with pcDNA (*lanes 1* and *3*) or a plasmid expressing Gag (*lanes 2* and *4*) were subjected to IP (1 mg of total protein) using anti-p24 antibody. 20 µg of total protein (*input*) and resuspended beads were analyzed western blot with anti-p24, anti-RPL7 and anti-GAPDH antibodies revealed by protein A-HRP. RPL7 signal was observed in *lane 4* showing that RPL7 was captured by Gag. In contrast, no RPL7 signal was observed in the pcDNA tranfected cells (*lane 3*) or in the assay where the beads were incubated in absence of anti-p24 antibody (*lane 5*). **c** Flag–RPL7 co-precipitates Gag-eGFP. HeLa cells were transfected with pcDNA (*lanes 1* and *5*), Gag-eGFP (*lanes 2* and *6*), Flag–RPL7 (*lanes 3* and *7*) or co-transfected with Flag–RPL7 and Gag-eGFP (*lanes 4*, *8* and *9*). IP was performed with 1.5 mg of total protein with a mouse anti-Flag antibody. 20 µg of *input* (*lanes 1–4*) and IPs (*lanes 5–8*) were resolved by 10 % SDS-PAGE and analyzed by immunostaining using an antibody directed against Flag to detect RPL7, eGFP to detect Gag, and GAPDH as a loading control. RPL7 is able to co-immunoprecipitate Gag-eGFP in HeLa cells (*lane 8*). No unspecific binding between Flag–RPL7 and beads was observed in the control without anti-flag antibody (*lane 9*)

The interaction between RPL7 and Gag was identified by mass spectroscopy [28, 29], sedimentation assay [30] and a high throughput two-hybrid screen (R. Benarous, personal communication). To confirm this interaction in a pairwise two-hybrid analysis, a cDNA coding for the full length RPL7 was cloned in fusion with the Gal4p-AD. As seen in Fig. 1a, diploid yeast cells expressing Gal4AD-RPL7 and Gal4BD-Gag had the capacity to grow on the selective medium lacking histidine. Controls consisting in the co-expression of either Gal4AD-RPL7 and Gal4AD, Gal4AD and Gal4BD-Gag were unable to grow in the same conditions indicating that the Gal4 activation observed was not due to the transactivation of one of the construct alone (Fig. 1a). It is worth noting that interactions were not tested with Gag fused to Gal4AD, due to its toxicity for the yeast *S. cerevisiae*.

To confirm the Gag–RPL7 interaction found by two-hybrid analysis, we carried out co-immunoprecipitation (co-IP) experiments using an anti-CA (anti-p24) antibody (Fig. 1b). After HeLa cell transfection of DNA expressing Gag, the cell extracts were analyzed for the expression of Gag (Fig. 1b, lane 2) and endogenous RPL7 (Fig. 1b, lanes 1 and 2) using monoclonal anti-p24 and polyclonal anti-RPL7 antibodies, respectively (Input). Then, equal quantities of cell extract were incubated with mouse anti-p24 antibodies and protein A-beads. The immunopurified material was analyzed by western blot using mouse anti-p24 and rabbit anti-RPL7 antibodies (Fig. 1b, IP). As shown in Fig. 1b, RPL7 was present together with Gag (Fig. 1b, lane 4) but absent from the mock cell lysate (Fig. 1b, lane 3) or when immunoprecipitation was performed without anti-p24 (Fig. 1b, lane 5).

To confirm this result, we performed a reverse experiment. To this end, HeLa cells were transfected with a plasmid expressing Flag–RPL7 alone or with a plasmid expressing Gag-eGFP. HeLa cell lysates were analyzed by SDS-PAGE followed by western blot for Flag–RPL7 and Gag-eGFP expression using anti-Flag (Fig. 1c, lanes 3 and 4) and anti-eGFP antibodies (Fig. 1c, lanes 2 and 4), respectively. In parallel experiments, cell lysates were incubated with anti-Flag antibody to immunoprecipitate RPL7 (Fig. 1c, lanes 7 and 8). In the presence of immunoprecipitated RPL7, a specific Gag signal was observed with the anti-eGFP antibody (Fig. 1c lane 8). Interestingly, all controls failed to precipitate Gag (Fig. 1c, lanes 5, 6, 7 and 9) confirming a specific interaction between Gag and RPL7.

### RPL7 interacts with Gag in HIV infected cells and is incorporated into virions

**Fig. 2:**
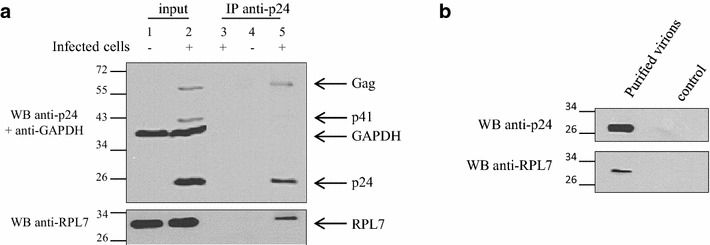
Interaction of HIV-1 Gag and RPL7 in infected cells and virion incorporation of RPL7. **a** Interaction of HIV-1 Gag and RPL7 in infected cell. Cell lysates of naïve CEM-SS (*Lanes 1* and *4*) or of HIV-1 LAI infected CEM-SS (*lanes 2* and *4*) were subjected to IPs 3 days post infection using anti-p24 antibody and protein A beads. The IP was followed by western blotting with anti-p24 and anti-RPL7 antibodies revealed by protein A-HRP and anti-rabbit HRP conjugate, respectively. Additionally, the total amount of protein loaded on the gel was controlled by anti-GAPDH antibody (*input*, *lanes 1* and *2*). A bead control using the infected cell lysate in the absence of p24 antibody was also performed (*lane 3*), confirming the specific binding of Gag and its processed forms (p24, p41, p55). **b** RPL7 is incorporated into HIV-1 particles. HIV-1 BaL virus stock produced on PHA-activated PBMC were purified and concentrated before been analyzed by western blotting using anti-p24 and anti-RPL7 antibodies. The control represents the purified supernatant from mock-infected PBMC using the same purification protocol

To analyze the Gag–RPL7 interaction in a viral context, CEM-SS cells were infected with HIV-1 LAI (CXCR4 strain). After 4 days, flow cytometry analysis showed that 62 % of the cells were infected (data not shown) [40]. Equal amounts of lysate from infected or naïve cells were analyzed by SDS-PAGE and by western-blotting using an anti-p24 antiserum. As seen in Fig. 2a (input), Gag and processed Gag (p41 and p24) were detected in the lysate of infected cells (lane 2) but not in that of naïve cells (lane 1). Meanwhile, these lysates were incubated with anti-p24 antibody and Gag and processed Gag were immunoprecipitated from infected cells lysates (Fig. 2a, lane 5) but not from naïve cell lysates (Fig. 2a, lane 4). In addition, as a control, the infected cell lysate was incubated with protein A-beads without anti-p24 antibody and the absence of signal in lane 3 (Fig. 2a) indicated that neither Gag nor RPL7 had nonspecific interactions on the beads. Interestingly, incubation of this membrane with an antibody directed against RPL7 revealed that endogenous RPL7 was co-immunoprecipitated by Gag in such infected cells (Fig. 2a, lane 5).

In order to test whether the interaction between Gag and RPL7 promotes the incorporation of RPL7 into a more relevant physiologically model of virions, HIV-1 BaL produced in primary cells was purified by a gel exclusion method [40, 41]. RPL7 was detected in these purified HIV-1 particles (Fig. 2b) while no RPL7 was found in the supernatant of naïve primary cells (Fig. 2b, control). Taken together, these results show that RPL7 and Gag interact in a viral context and that RPL7 can be recruited into infectious HIV-1 particles.

To look for the possible role of the Gag–RPL7 interaction in HIV-1 replication, siRNA directed against RPL7 was transfected and found to induce a large decrease of RPL7 protein (data not shown). Nevertheless, other proteins such as actin, RPS14 and nucleolin were impacted indicating that silencing of RPL7 has a negative effect on the translation machinery [36]. Therefore, due to these limitations, we could not evidence any clear impact of the Gag–RPL7 interaction on HIV-1 replication.

### NC zinc-fingers are key determinants for the Gag–RPL7 interaction

**Fig. 3:**
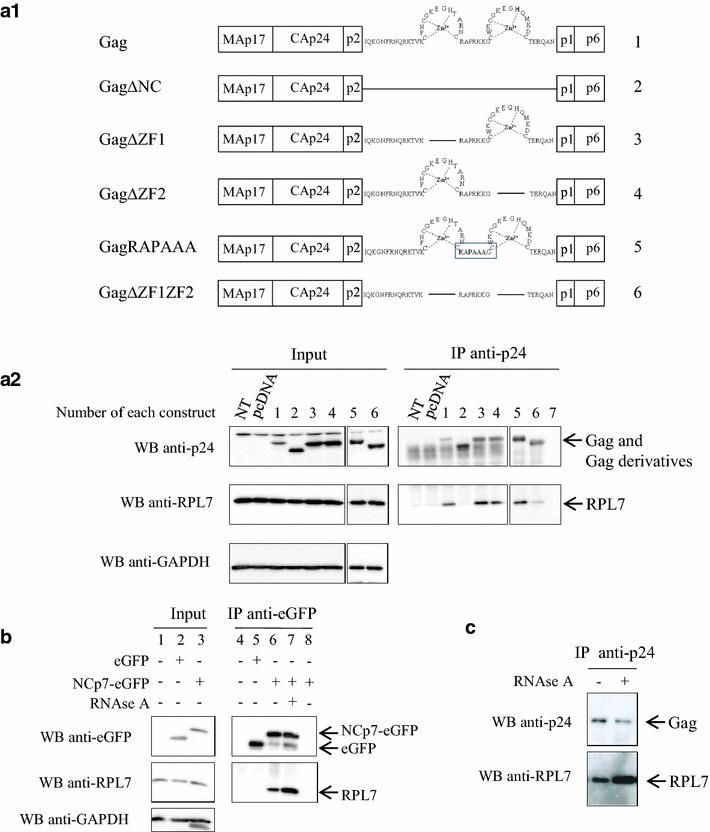
The NC domain of Gag mediates Gag–RPL7 interaction in an RNA independent manner. **a1** Gag and truncated Gag constructs used in the present study. Numbers on the right correspond to numbers of *lanes* of **a2**. **a2** The NC domain of Gag is important for the RPL7–Gag interaction. HeLa cells were not transfected (NT) or transfected with pcDNA or plasmids encoding either Gag (construct 1), Gag∆NC (construct 2), Gag∆ZF1 (construct 3), Gag∆ZF2 (construct 4), GagRAPAAA (construct 5) and Gag∆ZF1ZF2 (construct 6). IP was performed with 1 mg of total protein and with an anti-p24 antibody. 20 µg of cell lysate (*input*) or IP resuspended samples were resolved by 10 % SDS-PAGE and analyzed by immunoblotting using antibodies against p24, RPL7 and GAPDH revealed by protein A-HRP, anti-rabbit HRP conjugate or anti-mouse HRP conjugate, respectively. Nonspecific binding was not observed in the control without antibody (*lane 7*). **b** NCp7-RPL7 interaction is RNA independent. Cell lysate from HeLa cells transfected with empty pcDNA (*lanes 1, 4*) or plasmids coding for eGFP (*lanes 2, 5*) or NCp7-eGFP (*lanes 3, 6*). Cell lysates were immunoprecipitated with an anti-eGFP and the immunoprecipitated material was examined by western blot using anti-eGFP, RPL7 and GAPDH antibodies. *Lane 7* cell lysates expressing NCp7-eGFP were treated with RNase before immunoprecipitation. *Lane 8* cell lysate expressing NCp7-eGFP was incubated with protein A beads but without anti-eGFP antibody. **c** Gag–RPL7 interaction is RNA independent. Cell lysate from HeLa cells transfected with Gag was treated with RNase. After RNase treatment, cell lysate was immunoprecipitated with anti-p24 antibody, and the immunoprecipitate was analysed by western blot using anti-p24 and anti-RPL7 antibodies

In order to further map the GagNC determinants responsible for the interaction with RPL7, co-IP assays were performed using a number of HIV-1 Gag proteins mutated in their NC domain (Fig. 3a1). To investigate the role of the NC zinc fingers, Gag proteins where either one of the two zinc fingers was removed (GagΔZF1 and GagΔZF2) were compared to a deletion mutant missing the entire NC domain (GagΔNC). To determine the role of the basic sequences surrounding the CCHC motifs, both zinc fingers were deleted (GagΔZF1ΔZF2) or the ^32^RKK motif was substituted for AAA (GagRAPAAA). Expression of these Gag constructs was monitored by western blotting (Fig. 3a2, input). In parallel experiments, Gag and Gag derivatives were immunoprecipitated with anti-p24 antibody and membranes were immunoblotted with anti-p24 or anti-RPL7 antibodies followed by an incubation with protein A-HRP conjugate (Fig. 3a2, IP). GagNC mutants were immunoprecipitated (Fig. 3a2, lanes 1–6) with a higher efficiency as compared to the wild-type Gag protein (Fig. 3a2, lane 1), probably as a result of the masking of Gag epitopes due to the stronger ability of wild-type Gag to oligomerize, as compared to the Gag mutants [42–44]. These membranes were subsequently immunoblotted with the anti-RPL7 antibody, showing that endogenous RPL7 was captured by Gag, GagΔZF1, GagΔZF2 and GagRAPAAA (Fig. 3a2, lanes 1, 3, 4 and 5, respectively) at similar levels. In sharp contrast, RPL7 was poorly immunoprecipitated by Gag lacking either the entire NC domain or both zinc fingers (Fig. 3a2, lanes 2 and 6, respectively). Taken together, these results indicate that the ZFs but not the flanking basic sequences are key determinants for the interaction with RPL7.

Finally, a co-IP assay was carried out on cells expressing eGFP and NCp7-eGFP using an anti-GFP serum (Fig. 3b, input, lane 2 and 3). Endogenous RPL7 was co-eluted with NCp7-eGFP (Fig. 3b, lane 6) but not with the eGFP control (Fig. 3b, lane 5), thus confirming the two-hybrid screen (Fig. 1a). Taken together, these results indicate that NC, either in the form of GagNC or as mature NCp7, interacts with endogenous RPL7.

### Gag–RPL7 interaction is RNA independent

The NC domain of Gag and RPL7 are RNA binding proteins [34, 35, 45]. To determine the importance of RNA for the Gag–RPL7 interaction, lysates of cell expressing Gag and endogenous RPL7 were treated with RNase prior to IP with an anti-p24 antiserum. After RNase treatment, Gag was immunoprecipitated and detected by anti-p24 antibody (Fig. 3c, upper membrane). Then membranes were incubated with anti-RPL7 antibody and the endogenous protein was detected (Fig. 3c, lower membrane). Interestingly, the detection of RPL7 in the two samples shows that the RPL7 remains associated with Gag upon RNase treatment indicating that RNA may not be involved in the Gag–RPL7 interaction. Intriguingly, in a reproducible manner, the RPL7 signal was found to increase upon RNase-treatment (Fig. 3c) despite equal quantity of Gag immunoprecipitated on beads. To control the RNAse activity, RNA extraction by phenol–chloroform was performed on an aliquot of cell lysate containing RNAse. As seen in Additional file 1: Fig. S1, no RNA was detected in presence of RNAse while a smear of RNA was obtained without treatment. In addition, similar experiments were carried out with cells expressing NCp7-eGFP. As indicated (Fig. 3b, lane 7) NCp7-eGFP was able to precipitate endogenous RPL7 in presence of RNAse. Taken together, these results show that the interaction between Gag and RPL7 is probably not relying on a RNA template.

### The Gag–RPL7 interaction is independent from Gag-membrane interaction or Gag–Gag oligomerization

**Fig. 4:**
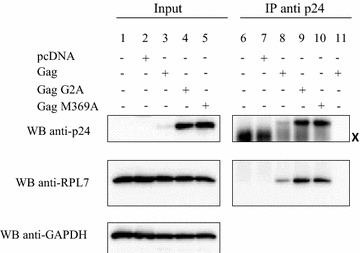
The Gag–RPL7 interaction is independent on Gag myristoylation and oligomerization. Lysates from HeLa cells transfected with pcDNA (*lanes 2, 7*) or plasmids coding for Gag (*lanes 3, 8* and *11*), Gag-G2A (*lanes 4, 9*) and Gag-M369A (*lanes 5, 10*). Control: lysate from non-transfected HeLa cells (*lanes 1, 6*). The lysates were subjected to IP (1 mg of total protein) using anti-p24 antibody followed by western blot where 20 µg of total protein (*input*) and resuspended beads were analyzed with anti-p24, anti-RPL7 and anti-GAPDH antibodies revealed by protein A-HRP, anti-rabbit HRP conjugate or anti-mouse HRP conjugate, respectively. All Gag proteins (WT or mutants) interact with endogenous RPL7 (*lanes 8–10*). Neither Gag nor RPL7 bands were observed in non-transfected cells (*lane 6*), in pcDNA transfected cells (*lane 7*), or in the control of beads without anti-p24 (*lane 11*). X Heavy chain of anti-p24 used to IP Gag and Gag derivatives

During assembly, newly made Gag molecules are progressively anchored onto the internal leaflet of the plasma membrane via the Matrix N-terminus and its myristate group at position 2. The G2A mutation impairs Gag membrane binding [46, 47], causing its accumulation in the cytoplasm [48, 49]. The absence of Gag myristoylation had no effect on the ability of Gag to immunoprecipitate RPL7 (Fig. 4, lane 9) indicating that Gag–RPL7 interaction does not require membrane association. Taking into account that G2A mutation also impairs, at least in part, Gag–Gag interaction [43, 44] this result indicates that Gag multimerization is probably not essential for the Gag–RPL7 interaction. In agreement with this conclusion, a Gag mutated in the capsid (M369A) with a severe defect in Pr55^Gag^ assembly [42, 44, 50–52] was found to immunoprecipitate RPL7 as efficiently as the wild-type Gag (Fig. 4, lane 10). Taken together these data show that the Gag–RPL7 interaction does not rely on the association of Gag with the plasma membrane and its oligomerization.

### Major RPL7 determinants for the interaction with HIV-1 Gag

**Fig. 5:**
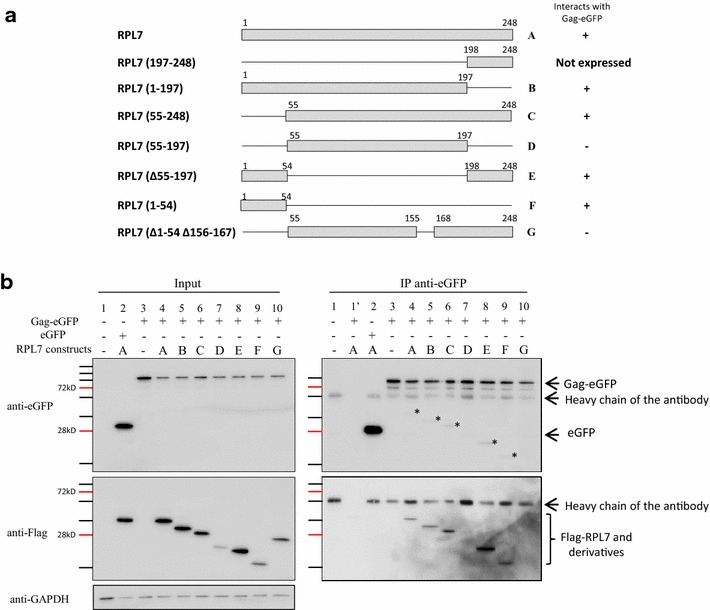
Mapping of the Gag interaction domains in RPL7. **a** Scheme of Flag–RPL7 and its mutants: Flag–RPL7 mutants used in the present study are presented with a *grey rectangle* (RPL7 sequence conserved in the constructs) and with a *black line* (RPL7 sequence deleted in the constructs). *Letters* correspond to each construct and are used in **b**. On the right, (+) and (−) report the ability of each construct to be co-precipitated with Gag-eGFP. **b** Identification of Flag–RPL7 interaction with Gag-eGFP by immunoprecipitation experiments. HeLa cells were either not transfected (*lane 1*), or co-transfected with plasmids expressing eGFP and Flag–RPL7 (**a**, *lane 2*), Gag-eGFP alone (*lane 3*) or Gag-eGFP combined with Flag–RPL7 A (*lane 4*) or Flag–RPL7 B (*lane 5*), Flag–RPL7 C (*lane 6*), Flag–RPL7 D (*lane 7*), Flag–RPL7 E (*lane 8*), Flag–RPL7 F (*lane 9*), Flag–RPL7 G (*lane 10*). IP was performed with 1 mg of total protein and with mouse anti-eGFP antibody. 20 µg of total protein (*input*) and resuspended beads were resolved by 10 % SDS-PAGE and analyzed by immunoblotting using antibody against Flag followed by anti-mouse HRP conjugate. Membranes were stripped and re-blotted using mouse anti-eGFP antibody followed by anti-mouse HRP conjugate. *Lane 1*′ corresponds to beads without antibody. *Asterisk* residual signal of the secondary anti-mouse interacting with primary anti-Flag antibody

To map the RPL7 determinants involved in Gag interaction, we used a panel of deletion mutants (Fig. 5a). The design of these mutants is based on the previously reported roles of the different domains of RPL7. Constructs C, E and G contain RPL7 C-terminus which interacts with the plasma membrane (PM) and RNA [34, 53]. Constructs B, E and F contain the RPL7 N-terminus which also interacts with nucleic acids and possesses a leucine zipper that promotes RPL7 self-oligomerization [54]. To discriminate the importance of each terminus with the central region of RPL7, a construct was designed harboring only the central domain (RPL7 D) and compared with a construct containing both the N- and C- termini (RPL7 E). Also, to underline the importance of RPL7 3-D structure [32, 55], a partial deletion in the central domain of RPL7 C was designed (RPL7 G). The immunoprecipitation of the RPL7 mutants was tested using anti-eGFP antibodies on HeLa cells expressing Gag-eGFP. Each lysate tested had an equal level of Gag-eGFP expression (Fig. 5b, input). Moreover, RPL7 (Fig. 5b, input, lanes 2 and 4) and RPL7 B, C, E and G (Fig. 5b, input, lanes 5, 6, 8 and 10) were expressed at a similar level except for RPL7 D and F (Fig. 5b, input, lanes 7 and 9). As shown in Fig. 5b (IP), RPL7 B, E and F (lanes 5, 8 and 9) containing the N-terminus and RPL7 C containing the C-terminus (lane 6) were immunoprecipitated by Gag and visualized by anti-Flag (IP).This result points to the importance of both the N- and C-termini of RPL7 for the interaction with Gag. Indeed, despite the low expression level when both N- and C- termini were removed (RPL7 D, input, lane 7), this construct was not immunoprecipated by Gag (IP, lane 7) suggesting that the central globular domain of RPL7 is dispensable for Gag interaction. In addition, there are likely other RPL7 domains involved in the interaction between RPL7 and Gag since deletion of the 155–168 sequence of construct C (RPL7 G) prevented Gag-mediated co-precipitation of RPL7 (Fig. 5b, IP, lane 10). However, this highly basic domain is located at the surface of the protein in a turn linking two α helices and the absence of interaction could result from a global change in the protein structure [32, 55]. Thus, our data show that the two terminal domains of RPL7 (residues 1–54 and 198–248) are required for Gag–RPL7 interaction.

### RPL7, Gag and Gag–RPL7 mixture have DNA/RNA chaperone activity

**Fig. 6:**
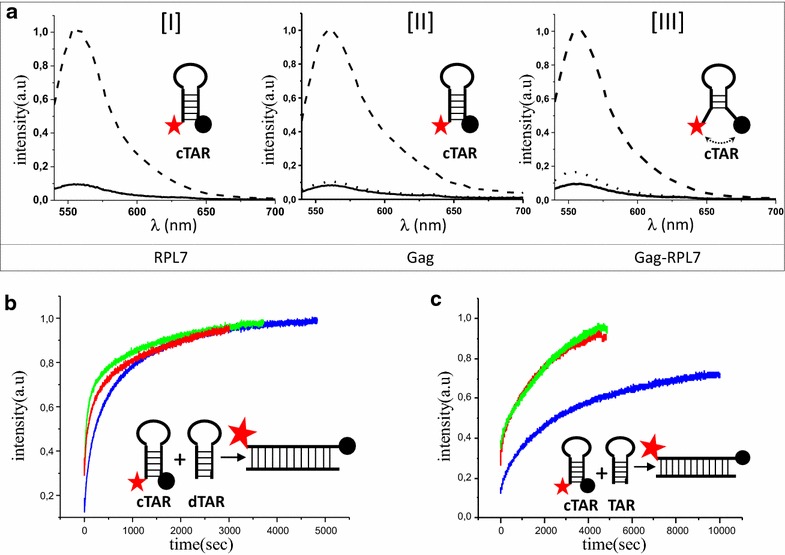
DNA and RNA annealing activity of RPL7. **a** Emission spectra of Rh6G-cTAR-Dabcyl. (*I*) Emission spectra of 10 nM Rh6G-cTAR-Dabcyl (*black line*) with 10 nM RPL7 (*dotted line*) or with 100 nM dTAR + 100 nM RPL7 after completion of the annealing reaction (*dashed line*).* Inset* scheme of closed doubly labelled cTAR:* red star* corresponds to Rh6G and* black circle* of Dabcyl. (*II*) Emission spectra of 10 nM Rh6G-cTAR-Dabcyl (*black line*) with 10 nM Gag (*dotted line*) or with 100 nM dTAR + 100 nM Gag after completion of the annealing reaction (*dashed line*). *Inset* scheme of closed doubly labelled cTAR. (*III*) Emission spectra of 10 nM Rh6G-cTAR-Dabcyl (*black line*) in the presence of 10 nM of a mixture of 5 nM Gag and 5 nM RPL7 (*dotted line*) or with 100 nM dTAR + 100 nM of the mixture of 50 nM Gag and 50 nM RPL7 after completion of the annealing reaction (*dashed line*). *Inset* scheme of doubly labelled cTAR with an *arrow* showing a slight fraying of cTAR. **b** Real time monitoring of cTAR-dTAR annealing. Kinetic traces for the reaction of 10 nM doubly labeled cTAR with 100 nM non-labeled dTAR in the presence of 100 nM of RPL7 (*red trace*) or 100 nM of Gag (*blue trace*) or 100 nM of a mixture containing 50 nM of Gag and 50 nM of RPL7 (*green trace*). Each *curve* corresponds to a single assay but is representative of four independent measurements. All assays were fitted by a bi-exponential equation and the values of the kinetic rate constants were reported in Table 1. *Inset* describes the scheme of cTAR-dTAR annealing. Formation of the duplex increases the distance between Rh6G and Dabcyl and thus restores Rh6G emission. **c** Real time monitoring of cTAR-TAR annealing. Kinetic traces for the reaction of 10 nM doubly labeled cTAR with 1 µM of non-labeled TAR in the presence of 1 µM of RPL7 (*red trace*) or 1 µM of Gag (*blue trace*) or 1 µM of a mixture containing 500 nM of Gag and 500 nM of RPL7 (*green trace*). Each *curve* corresponds to a single assay but is representative of four independent measurements. All traces were fitted by a bi-exponential equation and values of the kinetic rate constants were reported in Table 2. *Inset* describes the scheme of cTAR-TAR annealing

Next, we investigated the nucleic acid annealing activity of RPL7, Gag, and RPL7 and Gag together using purified proteins (Additional file 2: Fig. S2). The annealing activity of RPL7 was examined using an in vitro assay based on the annealing of dTAR, the DNA equivalent of the HIV-1 transactivation responsive element (TAR) with its complementary sequence cTAR DNA labeled by Rh6G and Dabcyl at its 5′ and 3′ ends, respectively [19, 56–60]. As shown in Fig. 6a (panel I, black curve), the emission of 10 nM of free labelled cTAR is low as a result of the close proximity of cTAR ends inducing a strong fluorescence quenching of Rh6G by the Dabcyl group [61]. Next, addition of 1 equivalent of RPL7 to this solution did not induced any increase in Rh6G fluorescence emission (Fig. 6a, panel I, dotted line), suggesting that, in contrast to NCp7 [62, 63], RPL7 is unable to melt the lower half of the cTAR stem (panel I, insert). Addition of a mixture of 100 nM of RPL7 and 100 nM of non-labeled dTAR to obtain pseudo-first order conditions induced a sevenfold increase of Rh6G emission (Fig. 6a, panel I, dashed line and Table 1). This increase results from the annealing of doubly labelled cTAR to dTAR and can be monitored in real time (Fig. 6b, red curve). The plateau, which corresponds to the total annealing of cTAR and dTAR into an extended duplex (ED) (Fig. 6b, insert), was completed in ~2000 s, while more than one day was needed in the absence of protein [57], indicating that RPL7 is endowed with a potent nucleic acid annealing activity. This kinetic curve was fitted by using a bi-exponential function with *k*_*obs1*_ = 10.6(±0.8) × 10^−3^ s^−1^ and *k*_*obs2*_ = 10.2(±0.3) × 10^−4^ s^−1^ (Table 1). To further confirm the nucleic acid annealing activity of RPL7, dTAR was substituted for TAR RNA. Full annealing of cTAR with 1 µM of TAR was obtained in 3500 s with *k*_*obs1*_ = 44(±1) × 10^−4^ s^−1^ and *k*_*obs2*_ = 39(±2) × 10^−5^ s^−1^ (Fig. 6c, red curve, Table 2), indicating that RPL7 is also able to promote annealing with RNA sequences, but less efficiently than the corresponding DNA sequences. A similar difference in efficiency as a function of the nature of the nucleic acid was also reported for NCp7 and was attributed to the higher stability of the RNA as compared to the DNA sequences [57]. In conclusion, our data indicate that, by analogy to ribosomal proteins L1 and L19 from *E. coli* [38], the human RPL7 is endowed with efficient nucleic acid annealing activities.

**Table 1:** Kinetic parameters for the annealing of cTAR to dTAR in the presence of Gag, RPL7 and the mixture of the two proteins

	RPL7	Gag	Gag + RPL7
a	0.58 ± 0.01	0.46 ± 0.01	0.80 ± 0.01
k_obs1_ (s^−1^)	(10.6 ± 0.8) × 10^−3^	(5.3 ± 0.4) × 10^−3^	(14 ± 0.7) × 10^−3^
k_obs2_ (s^−1^)	(10.2 ± 0.3) × 10^−4^	(8.2 ± 0.8) × 10^−4^	(18 ± 1) × 10^−4^
If/Io	6.5 ± 0.6	6.8 ± 0.5	7.2 ± 0.2

**Table 2:** Kinetic parameters for the annealing of cTAR to TAR in the presence of Gag, RPL7 and mixture of two proteins

	RPL7	Gag	Gag + RPL7
a	0.41 ± 0.04	0.22 ± 0.028	0.67 ± 0.01
k_obs1_ (s^−1^)	(44 ± 1) × 10^−4^	(13.5 ± 0.7) × 10^−4^	(57 ± 1) × 10^−4^
k_obs2_ (s^−1^)	(39 ± 2) × 10^−5^	(20.5 ± 0.7) × 10^−5^	(47 ± 1) × 10^−5^
If/Io	8.2 ± 0.2	6.1 ± 0.2	8.45 ± 0.3

Next, we investigated the annealing activity of Gag in similar conditions. Addition of 10 nM of Gag to 10 nM of doubly labelled cTAR induced a limited increase in the emission of Rh6G (Fig. 6a, panel II, dotted line), suggesting that Gag has marginal destabilization activity in these conditions. Addition of 100 nM of Gag and 100 nM of non-labeled dTAR resulted in a large increase in Rh6G emission (Fig. 6a, panel II, dashed curve) that can be monitored in real time (Fig. 6b, blue curve). The annealing reaction took more than 4000 s to form the final ED providing values of *k*_*obs1*_ = 5.3(±0.4) × 10^−3^ s^−1^ and *k*_*obs2*_ = 8.2(±0.8) × 10^−4^ s^−1^ (Table 1). When this experiment was performed with 1 µM of TAR, the time to complete the reaction was longer (8000 s) and both *k*_*obs1*_ and *k*_*obs2*_ were slower (Fig. 6c, blue curve and Table 2). For both cTAR-dTAR and cTAR-TAR systems, the k_obs1,2_ values were about two to threefold higher with RPL7 as compared to Gag, indicating that RPL7 possesses a more potent nucleic acid annealing activity than Gag in the present conditions.

Finally, we investigated the chaperone activity of a Gag and RPL7 mixture. Interestingly, addition of 10 nM of Gag/RPL7 mixture (corresponding to 5 nM of Gag and 5 nM of RPL7) induces a ~2.5-fold increase of the doubly labelled cTAR fluorescence (Fig. 6a, panel III, dotted line), indicating that both proteins together can slightly destabilize the cTAR stem. Moreover, full annealing of cTAR with dTAR (Fig. 6b, green trace) or TAR (Fig. 6c, green trace) in presence of 100 nM of Gag/RPL7 mixture (corresponding to 50 nM of Gag and 50 nM of RPL7) was complete in less than 2000 and 4000 s, respectively for dTAR and TAR. Importantly, the k_obs1,2_ values with the Gag/RPL7 mixture is up to 1.5 to 3-fold higher than for the individual proteins in the case of the cTAR-dTAR system and is up to 1.5 to 4-fold higher for the cTAR-TAR system (Tables 1, 2). The substantial increase in both the nucleic acid destabilization and annealing activity of the protein mixture as compared to each protein indicates that the two proteins act in concert to promote nucleic acid annealing. These data suggest that Gag can improve its nucleic acid chaperone activity through the concerted activity of RPL7.

## Discussion

In HIV-1 infected cells, virus assembly is orchestrated by the structural polyprotein precursor Gag, but where and how assembly initially takes place is poorly understood. The current view stipulates that Gag binds specific motifs in the 5′ UTR of the FL viral RNA, which in turn causes its dimerization. The dimeric RNA genome is then thought to act as a platform to recruit Gag molecules via their NC domain [4, 7].

In order to better understand the initial step of Gag assembly, we wanted to identify cellular co-factors interacting with the NC domain of Gag. Among the possible candidates, RPL7 was shown to interact with both Gag and NCp7 [28]. Using yeast two hybrid and co-IP experiments, we confirmed that RPL7 interacts with Gag and NCp7 (Fig. 1) and that RPL7 can be incorporated in infectious particles (Fig. 2), together with other ribosomal proteins [64, 65]. Also, RPL7a can be incorporated into the virus as part of the Staufen1 ribonucleoprotein complex [66]; but RPL7 and RPL7a are different proteins since they exhibit only limited homology. As reported in Figs. 3 and 5 the Gag–RPL7 interaction relies on the two zinc fingers of GagNC and on both the N- and the C-terminal regions of RPL7. Data also indicates that the interaction of Gag with RPL7 is marginally dependent on RNA (Fig. 3) as well as on Gag oligomerization and its interaction with the plasma membrane (Fig. 4). Taken together, formation of the Gag–RPL7 complex seems to rely on direct protein–protein interaction or involves a still unidentified factor independent from the RNA-driven Gag assembly.

Noticeably, the amount of co-immunoprecipitated RPL7 with Gag was clearly increased upon RNase treatment (Fig. 3c), suggesting that RNA could outcompete the Gag–RPL7 interaction. Since the NC domain of Gag, as well as the N- and C- termini of RPL7 interact with RNA [34, 35], this suggests an overlap between the GagNC-RPL7 interacting domains and nucleic acid binding domains. Further studies on the tripartite complex Gag–RPL7-RNA should clarify a potential role of RNA, either cellular or viral, to regulate Gag–RPL7 complex formation.

We also show that RPL7 promotes the annealing of the complementary cTAR to dTAR or to TAR sequences (Fig. 6b, c). This property for a ribosomal protein to exhibit nucleic acid chaperone activity has been previously reported for an *E*. *coli* protein [38, 39] but not for a mammalian ribosomal protein. This nucleic acid chaperoning activity of RPL7 leads us to propose that RPL7 could facilitate structural rearrangements of mRNA during translation. Meanwhile, we confirmed that Gag has a weak nucleic acid chaperone activity especially for RNA (Fig. 6c) [22–24], and that both proteins can act in concert to promote nucleic acid destabilization and annealing.

**Fig. 7:**
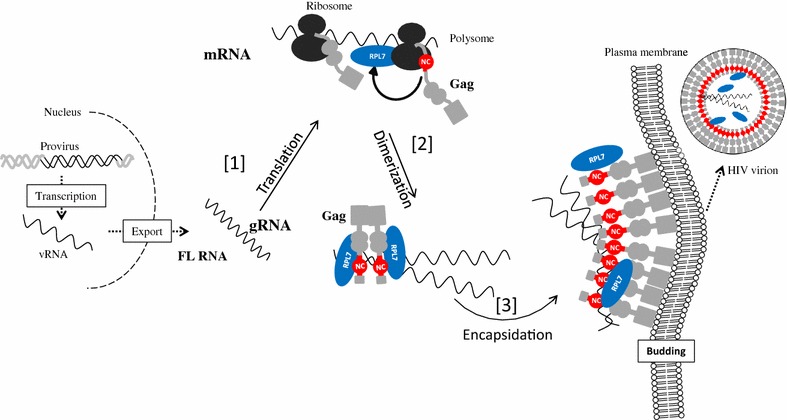
Proposed model for Gag mediated recruitment of RPL7 during HIV replication. Part of the late phase of HIV-1 replication is summarized in three steps, translation [1], dimerization of FL RNA [2] and its encapsidation [3]. Gag is in *grey*, the NC domain of Gag in *red* and RPL7 in *blue*

Thus, collectively, our data show that Gag interacts with RPL7, a cellular protein endowed with chaperone activity (Fig. 7). In the infected cell, when the unspliced HIV-1 FL RNA is exported from the nucleus to the cytoplasm and there transported to the virion assembly site by diffusion [67, 68] or by the microtubule/dynein pathway [69–72], it serves both as the gRNA and mRNA. As mRNA, it is recruited by the translational machinery to produce the Gag and GagPol polyproteins (Fig. 7—step 1) and as the gRNA it is selected by newly made Gag/GagPol. This latter specific interaction in turn causes its dimerization (Fig. 7—step 2) and tRNA^Lys, 3^ placement onto the genome (not shown in this scheme), concomittant with assembly (Fig. 7—step 3). However, the nucleic acid chaperone activity of GagNC is low (this study and [22–24, 73]) favoring the notion that Gag could recruit a cellular partner to facilitate these RNA annealing reactions. In line with this notion, the human RHA, also known as DHX9 was shown to participate in primer tRNA^Lys, 3^ annealing to the viral RNA [27]. Here, we go further proposing a model where Gag physically recruits RPL7 in an RNA independent manner to enhance its chaperone activity. Since the annealing activity of the Gag–RPL7 complex corresponds to the sum of the activities of the individual proteins (Fig. 6b, c, green curves), we propose that the complex might rapidly direct HIV-1 FL RNAs dimerization and primer tRNA annealing at the start of Gag assembly (Fig. 7—step 3). In addition, Gag and RPL7 were shown to inhibit translation through an unknown mechanism [35, 37, 74–77], possibly causing a functional switch from RNA translation to Gag assembly. This hypothesis is presently under investigation.

## Conclusion

Here we report that the cellular ribosomal protein RPL7 appears to be a Gag helper chaperoning factor possibly acting in concert with the nucleic acid chaperone activity of GagNC during assembly. Further experiments are needed to elucidate the function of Gag–RPL7 on the translation of mRNA and the regulation of the balance between FL RNA translation and encapsidation.

## Methods

### Two-hybrid system

Using the Gateway™ recombination technology (Life Technologies) a human RPL7 cDNA was cloned into pActII in fusion with the Gal4p Activation Domain (Gal4AD), Gag and NCp7 cDNA were cloned into pGBKT7 in fusion to the Gal4p DNA binding domain (Gal4BD). All pActII and pGBKT7 constructs were introduced into the *S. Cerevisiae* strain AH109 (MAT a, trp 1-901, leu2-3, 112, ura3-52, his3-200, Δgal4, Δgal80, LYS2: GAL1UAS-GAL1TATA-HIS3, GAL2UAS-GAL2TATA-ADE2, URA3: MEL1UAS-MEL1TATA-lacZ) or Y187 (MATα, ura3-52, his3-200, ade2-101, trp1-901, leu2-3, 112, gal4Δ, met–, gal80Δ, URA3::GAL1_UAS_-GAL1_TATA_-lacZ) respectively using a LiCl procedure [78]. The transformed cells were selected for Leu or Trp auxotrophy on minimal media plates (6.8 g L^−1^ YNB w/o amino acids (Sigma ref Y0626), 0.6 g L^−1^ of Drop OUT (ForMedium LTD, Hunstanton, England), 2 % glucose, 20 g L^−1^ Bacto-agar (Difco ref 214010). To carry out the two-hybrid test, yeast cells of each mating type transformed with the studied constructs were mated overnight and the diploids selected on a minimal medium depleted for Leu and Trp. The interaction between the two proteins tested was assayed by a 5-days growth of diploid yeasts on a minimal medium depleted for Leu, Trp and His.

### Plasmid DNA

The human-codon-optimized Pr55^Gag^ and pNL4-3^EGFP^ encoding plasmids were kindly provided by David E. Ott (National Cancer Institute at Frederick, Maryland) and Barbara Muller (Department of Infectious Diseases, Heidelberg). Pr55^Gag^ plasmid was used to obtain modified Gag proteins (Gag ΔZF1, GagΔZF2, GagΔZF1ΔZF2, GagRAPAAA and GagΔNC) by PCR-based site-directed mutagenesis following the supplier’s protocol as previously described (Thermo Scientific, F541) [79]. Construction of plasmids expressing Gag-eGFP and NCp7-eGFP were already described [79, 80]. The 3X Flag RPL7 was obtained by cloning the RPL7 (NCBI ref NM_000971.3) cDNA in frame with three copies of the Flag tag (DYKDDDDK) into a pCI-neo (Promega) backbone using the Gateway™ recombination technology (Life technologies). The integrity of all plasmid constructs was assessed by DNA sequencing (GATC Biotech, Germany).

### Cell culture and plasmid transfection

2 × 10^5^ HeLa cells (from ATCC, CCL-2 Amp, HeLa; Cervical Adenocarcinoma; Human) were cultured in 6-wells plate containing Dulbecco’s modified Eagle medium supplemented with 10 % fetal bovine serum (Invitrogen Corporation, Cergy Pontoise, France) and 1 % of an antibiotic mixture (penicillin/streptomycin DE17-602E: Lonza, Bal, Switzerland, DE16-602E) at 37 °C in a 5 % CO_2_ atmosphere. HeLa cells were transfected or co-transfected using jet PEI™ (Life Technologies, Saint Aubin, France).

### HIV purification and cell infection

CEM-SS cells [81] were infected with HIV-1 LAI. After 3 days of culture, the cells were splited into two batches to analyze the percentage of infected cells by intracellular p24 staining, using anti-HIV-1 core protein p24 (Ref RD-1, Beckman Coulter), and to measure the expression of p24 and RPL7 by flow cytometry and by western blot [40, 82].

To analyze the presence of RPL7, HIV-1 strain BaL primary isolate (subtype B, HIV-1 R5 strain, provided through the AIDS Research and Reference Reagent Program from Dr. S. Gartner; Department of Neurology, Johns Hopkins Hospital, Baltimore, MD, and Drs. M. Popovic and R. Gallo, Institute of Human Virology, University of Maryland Biotechnology Institute and Department of Microbiology, Baltimore, MD) was produced on PHA-activated peripheral blood mononuclear cell (PBMC). The virus stock was purified from cultured supernatant by a gel-filtration exclusion method on Sephacryl S-1000 Superfine (Amersham) columns as described previously [41, 82]. Supernatant of mock-infected cells was purified in parallel. The purified fractions were concentrated by 80-fold through a 100 kDa cut-off polyethersulfone filter (Centricon 80 Plus Biomax Filter; Millipore, Molsheim, France) before analysis by western blot.

### Immunoprecipitation and western blotting

HeLa cells were transfected and 24 h post-transfection, cells were harvested after trypsin treatment and resuspended in lysis buffer (10 mM Tris/HCl pH 7.5, 150 mM NaCl, 1 mM EDTA, 1 % NP40, 0.5 % SDS) supplemented with complete protease inhibitor cocktail (Roche Diagnostics GmbH). Cell lysates were cleared by centrifugation, and the supernatant corresponding to 1 mg of protein was incubated with primary antibody either mouse anti-p24 (Ref 6521 #24-4; AIDS Reagent Program, Division of AIDS, NIAID, NIH from Dr. Michael H. Malim), mouse anti-Flag (Sigma, F1804), or mouse anti-eGFP (Lifetech, A11120) for 2 h at 4 °C under continuous agitation. Protein A magnetic beads (Millipore, Pure Proteome, LSKMAGA10) were added for 90 min at 4 °C, and washed twice with ice-cold lysis buffer. Immunoprecipitated proteins (IP and Co-IP) and cell lysates (input) were analyzed by 10 % SDS-PAGE and membranes blotted either by mouse anti-p24 (Ref 6521 #24-4; AIDS Reagent Program, Division of AIDS, NIAID, NIH from Dr. Michael H. Malim), rabbit anti-Flag (Sigma, F1804), mouse anti-eGFP (Proteintech 66002-1), mouse anti-GAPDH (Millipore,MAB374) or rabbit anti-RPL7 (Abcam, ab72550) antibodies followed by anti-mouse HRP conjugate (Promega, W4021, 1:10,000) or anti-rabbit HRP (Promega, W401B) or by Protein A HRP (horseradish peroxidase, invitrogen, 10-1023).

For RNAse assay, the supernatant containing 1 mg of protein was incubated with a mixture containing 5 unit of RNAse A and 200 units of RNAse T1 (AM 2286) for 30 mn at room temperature. To verify RNAse activity, RNAs were extracted by addition of phenol–chloroform (v/v) using the kit “Tri Reagent Protocol” from sigma Aldrich (T9424). After extensive vortex and centrifuge at 12,000*g*, the supernatant was mixed with RNA loading dye (New England Biolabs, B0363S), loaded on a TAE (Tris Acetate EDTA) 1× 1 % agarose gel and visualized by Ethidium Bromide. Ladder was supplied from New England Biolabs (N3232S) (Additional file 1: Fig. S1).

### Protein purification

BL21 (DE3) cells were cultured in LB (Luria–Bertani) medium (1 % (w/v) peptone, 0.5 % (w/v) yeast extract, and 0.5 % NaCl). The medium was supplemented with kanamycin (50 µg mL^−1^) and chloramphenicol (25 µg mL^−1^) in order to purify HIV-1 Gag, and kanamycin (50 µg mL^−1^) and ampicillin (50 µg mL^−1^) to purify human RPL7. Recombinant protein production was performed by inoculating a single colony in 50 mL LB containing antibiotics, and cultured at 37 °C, overnight with shaking at 220 rpm. The overnight culture was used to inoculate 1 L LB containing antibiotics. The culture was grown at 37 °C, 220 rpm, until an absorbance at 600 nm of 0.6 was reached. Protein expression was induced by addition of 1 mM IPTG (Isopropyl β-d-1-thiogalactopyranoside) for 3 h at 37 °C or of 0.5 mM IPTG for 16 h at 18 °C for RPL7 or Gag, respectively. Bacteria were harvested and snap frozen in liquid nitrogen and stored at −80 °C. Both proteins were purified using previously described protocols [83, 84] and their purity was checked by polyacrylamide gel electrophoresis (Additional file 2: Fig. S2). Their concentration was measured from their absorbance at 280 nm using ε_RPL7_ = 27,000 L mol^−1^ cm^−1^ and ε_Gag_ = 63,000 L mol^−1^ cm^−1^.

### Monitoring nucleic acid annealing kinetics by fluorescence spectroscopy

Kinetic measurements were performed in pseudo first-order conditions by using 10 nM of cTAR labeled at its 5′ and 3′ ends by 6-carboxyrhodamine (Rh6G) and 4-(4′-dimethylaminophenylazo)benzoic acid (Dabcyl) with a 100 nM concentration of unlabeled dTAR (or 1 µM of TAR) as previously described [19, 85]. The reaction was monitored by recording the changes in the Rh6G fluorescence intensity at 555 nm (with excitation at 520 nm) with time. Proteins (Gag or RPL7) were added at a 1 molar ratio to each reactant separately and then, the reaction was initiated by mixing the protein-coated oligonucleotides together. The apparent rate constants *k*_obs_ and the amplitudes (a) were determined from the kinetic traces by including a dead-time correction *t*_0_ to take into account the delay between mixing the reactants and the start of the measurements. All fitting procedures were carried out with Origin™ 8.6 software based on nonlinear, least-square methods and the Levenberg–Marquardt algorithm. Emission spectra and kinetic traces were recorded with Fluorolog and FluroMax spectrofluorimeters (Jobin–Yvon Instruments, S.A. Inc.) equipped with a temperature-controlled cell compartment locked at 20 °C. All fluorescence intensities were corrected for buffer emission and lamp fluctuations. Experiments were performed at 20 °C in 50 mM Hepes, 150 mM NaCl, 1 mM DTT, 1 mM MgCl2, pH 7.4.

## Additional files

10.1186/s12977-016-0287-4 Control of RNAse activity. Cells were transfected with a plasmid expressing Gag. After trypsin treatment and addition of lysis buffer, cell lysate was incubated with RNAse cocktail containing RNAse A and T1. Then an aliquot was treated with phenol-chloroform to extract the RNAs and the supernatant was loaded on 1% agarose gel. 1: cell lysate incubated with RNAse cocktail. 2: cell lysate without addition of RNAse cocktail.
10.1186/s12977-016-0287-4 Coomassie-stained SDS–PAGE of the purified human RPL7 and HIV-1 wild type Gag. The molecular mass of the two proteins is 31 kDa and 57 kDa, respectively.

## Abbreviations

HIV-1: human immunodeficiency virus
RPL7: ribosomal protein L7
FL RNA: full length RNA
HRP: horseradish peroxidase
Gal4AD: Gal4p activation domain
Gal4BD: Gal4p DNA binding domain
eGFP: enhanced green fluorescent protein
SDS-PAGE: sodium dodecyl sulfate–polyacrylamide gel electrophoresis
IPTG: isopropyl β-d-1-thiogalactopyranoside
Rh6G: rhodamine 6G
Dabcyl: 4-(4-dimethylaminophenylazo)benzoyl
TAR: transactivation responsive element
dTAR: deoxyribonucleic equivalent of TAR
cTAR: complementary sequence of dTAR
ED: extended duplex
