# Supplementary figures and images for: Characterization of the interaction between the HIV-1 Gag structural polyprotein and the cellular ribosomal protein L7 and its implication in viral nucleic acid remodeling
Source: Retrovirology. 2016 Aug 11;13:54. doi: 10.1186/s12977-016-0287-4 (PMC4982112; doi:10.1186/s12977-016-0287-4)

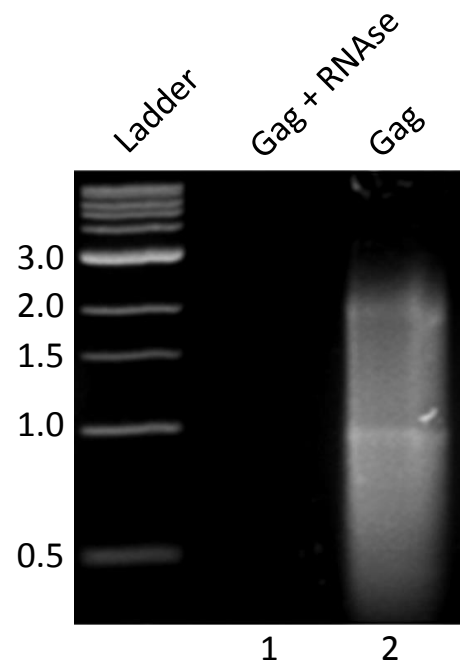

Figure S1

Supplement: Supplementary file 1 — 10.1186/s12977-016-0287-4 Control of RNAse activity. Cells were transfected with a plasmid expressing Gag. After trypsin treatment and addition of lysis buffer, cell lysate was incubated with RNAse cocktail containing RNAse A and T1. Then an aliquot was treated with phenol-chloroform to extract the RNAs and the supernatant was loaded on 1% agarose gel. 1: cell lysate incubated with RNAse cocktail. 2: cell lysate without addition of RNAse cocktail. [file 12977_2016_287_MOESM1_ESM.pdf]

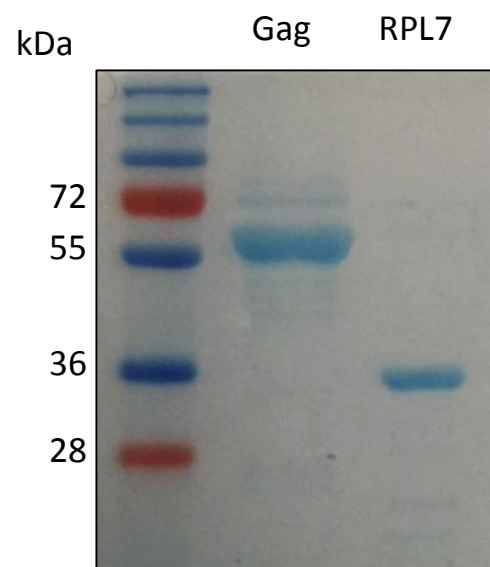

Figure S2

Supplement: Supplementary file 2 — 10.1186/s12977-016-0287-4 Coomassie-stained SDS–PAGE of the purified human RPL7 and HIV-1 wild type Gag. The molecular mass of the two proteins is 31 kDa and 57 kDa, respectively. [file 12977_2016_287_MOESM2_ESM.pdf]
